# American Medical Association

**Published:** 1851-07

**Authors:** 


### THE WESTERN JOURNAL.

THIRD SERIES.—VOL. VIII.—NO. I.

LOUISVILLE, JULY 1, 1851.

### AMERICAN MEDICAL ASSOCIATION.

The meeting of the American Medical Association at Charleston was entirely successful. The attendance was large, the exercises spirited, and the temper manifested by the members liberal and harmonious. In a social point of view, it is said to have been the most interesting meeting yet held. From the report which will be found in another part of this number, we infer that the amount of talent brought out by it was fully equal to that elicited by any previous meeting. Our impression is that these meetings have been increasing in earnestness, and that the conviction is growing stronger in the minds of the leading men of the profession, that the Association has force and value in it. We think we can discover in its last proceedings evidences of a determination, never shown in the same degree before, to render it effective. There is no longer any doubt respecting its perpetuity, and many who at first held back begin now to show a willingness to go to work and develope all its capacities of usefulness.

It will be seen, that the standing committees have been abolished, and that in future there are to be appointed in their stead committees for the investigation of special subjects. Upon the whole, we approve of the change and believe it will be found to impart a higher interest to the labors of the Association. Many of the reports heretofore made have been judicious and able and have embodied a mass of matter of great value, but in the nature of things such reports as are contemplated by the new provision must possess a freshness that cannot be given to stereotyped subjects. The topics proposed for reports to the next meeting appear to us to have been well chosen.

We regret that the effort of Dr. Drake to *popularize* the Association was not successful. We believe it would be better to bring in as many good members, by the method advocated by Dr. Drake, as possible, and to give to all such full privileges. It would increase the attendance and add new life to the meetings, and, what is of more importance, deepen the interest felt in the Associa.ion by the profession. We hope the proposed change in the constitution will yet be made.

If we were to except to anything done at the late meeting, it would be the tablet voted to the profession of Charleston for their extraordinary hospitality to the members of the Association. Not that the compliment was not fully merited; we can readily believe all that has been said of the warmth and cordiality with which the Association was welcomed and entertained by the physicians of that hospitable city; but the action prompted by feelings so praiseworthy, and so hard to resist in this case, is likely to become a precedent for future action, and may lead to unpleasant results. The next city will naturally vie with Charleston in acts of hospitality, and may be fortunate enough to surpass her munificent neighbor. A vote of thanks and a tablet must follow. Any one can see that this course long persisted in, would be productive of much inconvenience, and could not fail to impair the usefulness of the Association. But, in its migrations, it must occasionally visit places less opulent than Charleston and where the profession, with the best disposition in the world, could not treat it so sumptuously. If the tablet be voted in such a case, it becomes a mere empty form; if it is withheld, the distinction is invidious and there is danger that ill feeling will be engendered.

We are glad that the Association has expressed an opinion, which we hope will be authoritative, in regard to the attendance of students of medicine during the entire course of lectures. More than once we have adverted to this subject, and have earnestly deprecated the practice too common with students of quitting the lectures before the end of the term. Y.

